# Transcriptome analysis of the central nervous system of the mollusc *Lymnaea stagnalis*

**DOI:** 10.1186/1471-2164-10-451

**Published:** 2009-09-23

**Authors:** Z-P Feng, Z Zhang, RE van Kesteren, VA Straub, P van Nierop, K Jin, N Nejatbakhsh, JI Goldberg, GE Spencer, MS Yeoman, W Wildering, JR Coorssen, RP Croll, LT Buck, NI Syed, AB Smit

**Affiliations:** 1 Department of Physiology, University of Toronto, Toronto, Ontario M5S 1A8, Canada; 2 Terrence Donnelly Centre for Cellular and Biomolecular Research, University of Toronto, Toronto, Ontario M5S 3E1, Canada; 3 Department of Molecular & Cellular Neurobiology, Center for Neurogenomics and Cognitive Research, Neuroscience Campus Amsterdam, VU University, The Netherlands; 4 Department of Cell Physiology & Pharmacology, University of Leicester, Leicester LE1 9HN, UK; 5 Department of Biological Sciences, University of Calgary, Calgary, AB T2N 1N4, Canada; 6 Department of Biological Sciences, Brock University, St. Catharines, ON L2S 3A1, Canada; 7 School of Pharmacy & Biomolecular Sciences, University of Brighton, Brighton BN2 4GJ, UK; 8 Molecular Physiology, School of Medicine, and the Molecular Medicine Research Group, University of Western Sydney, NSW, Australia; 9 Departments of Physiology & Biophysics and Cell Biology & Anatomy, Hotchkiss Brain Institute, University of Calgary, Calgary, AB T2N 4N1, Canada; 10 Department of Physiology and Biophysics, Dalhousie University, Faculty of Medicine, Halifax, Nova Scotia B3H 1X5, Canada; 11 Departments of Cell and Systems Biology and Ecology and Evolutionary Biology, Toronto, ON M5S 3G5, Canada

## Abstract

**Background:**

The freshwater snail *Lymnaea stagnalis *(*L. stagnalis*) has served as a successful model for studies in the field of Neuroscience. However, a serious drawback in the molecular analysis of the nervous system of *L. stagnalis *has been the lack of large-scale genomic or neuronal transcriptome information, thereby limiting the use of this unique model.

**Results:**

In this study, we report 7,712 distinct EST sequences (median length: 847 nucleotides) of a normalized *L. stagnalis *central nervous system (CNS) cDNA library, resulting in the largest collection of *L. stagnalis *neuronal transcriptome data currently available. Approximately 42% of the cDNAs can be translated into more than 100 consecutive amino acids, indicating the high quality of the library. The annotated sequences contribute 12% of the predicted transcriptome size of 20,000. Surprisingly, approximately 37% of the *L. stagnalis *sequences only have a tBLASTx hit in the EST library of another snail species *Aplysia californica *(*A. californica*) even using a low stringency e-value cutoff at 0.01. Using the same cutoff, approximately 67% of the cDNAs have a BLAST hit in the NCBI non-redundant protein and nucleotide sequence databases (nr and nt), suggesting that one third of the sequences may be unique to *L. stagnalis*. Finally, using the same cutoff (0.01), more than half of the cDNA sequences (54%) do not have a hit in nematode, fruitfly or human genome data, suggesting that the *L. stagnalis *transcriptome is significantly different from these species as well. The cDNA sequences are enriched in the following gene ontology functional categories: protein binding, hydrolase, transferase, and catalytic enzymes.

**Conclusion:**

This study provides novel molecular insights into the transcriptome of an important molluscan model organism. Our findings will contribute to functional analyses in neurobiology, and comparative evolutionary biology. The *L. stagnalis *CNS EST database is available at .

## Background

**Figure 1:**
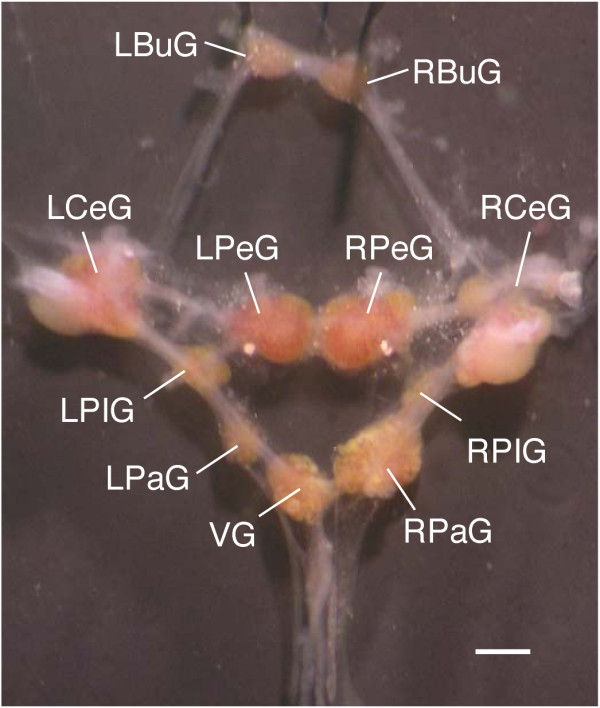
**The dissected central ring ganglia of *Lymnaea stagnalis***. LBuG and RBuG: left and right buccal ganglia; LCeG and RCeG: left and right cerebral ganglia; LPeG and RPeG: left and right pedal ganglia; LPIG and RPIG: left and right pleural ganglia; LPaG and RPaG: left and right parietal ganglia; VG: visceral ganglion. Scale bar: 1 mm.

The freshwater pond snail, *Lymnaea stagnalis *(*L. stagnalis, Linnaeus, 1758*), belongs to the phylum Mollusca (Gastropoda, Basommatophora, Pulmonata, Lymnaeidae). Similar to other gastropods such as the marine snail *Aplysia californica (A. californica) *and the terrestrial snail *Helix aspersa*, *L. stagnalis *has served successfully as a model for a wide spectrum of studies in molecular, cellular, and behavioral neurobiology. As compared to the mammalian brain with 10^11 ^neurons and *Drosophila melanogaster (D. melanogaster) *ganglia comprising 200,000 neurons, *L. stagnalis *has a relatively simple central nervous system (CNS) consisting of a total of ~20,000 neurons, many of them individually identifiable, organized in a ring of interconnected ganglia (Figure 1). Most neurons of the *L. stagnalis *CNS are large in size (diameter: up to ~100 μm), thus allowing electrophysiological dissection of neuronal networks that has yielded profound insights in the working mechanisms of neuronal networks controlling relatively simple behaviors such as feeding [1,2], respiration [3,4], locomotion [5], and reproduction [6,7]. Studies using the CNS of *L. stagnalis *as a model have also identified novel cellular and molecular mechanisms in neuronal regeneration [8-11], synapse formation [12-15], synaptic plasticity [16], learning and memory formation [17,18], the neurobiology of development [19-22] and aging [23-25], the modulatory role of neuropeptides [26-28], and adaptive responses to hypoxic stress [29-32]. Contrasting the large body of dedicated studies in neurobiology, the molecular analysis of genomic information of *L. stagnalis *is rather limited. The only available transcript sequence data set of *L. stagnalis *includes 1,320 expressed sequence tags (ESTs; derived from 771 different sequences) generated from *L. stagnalis *CNS libraries that were not normalized [33]. The lack of adequate transcriptome and genome information of *L. stagnalis *is currently a large drawback for the use of this species in functional and comparative molecular studies [34].

Mollusks have more than 100,000 extant species and comprise the second largest phylum after the Arthropods [35,36], indicating the phylum has been highly adaptive to environmental changes since its origin in the Cambrian period [35]. The gastropods are the largest group within the mollusca encompassing over 80% of the extant species [37]. However, in contrast to their abundance and importance in neurobiology, large-scale genomic information relating to neuronal function is limited to one species, *A. californica *[38]. There are two additional on-going genomic sequencing projects of mollusks: *Lottia gigantea *(*L. gigantean*; ) and *Biomphalaria glabrata *(*B. glabrata*; ); however, the genomic sequence information from these two species is currently not yet available. As *L. stagnalis *and *A. californica *genera are believed to have diverged over 600 million years ago, the sequencing of the CNS transcriptome from *L. stagnalis *holds significant promise for functional, evolutionary, comparative and environmental studies of Mollusks and other species.

We have carried out transcriptome sequencing of a normalized cDNA library constructed from the *L. stagnalis *CNS. This study provided 10,375 EST sequences, which cluster to 7,712 unique sequences. Despite a substantial fraction of the cDNAs being homologous to *A. californica *sequences in the existing known sequence database, data analysis revealed that the majority of the *L. stagnalis *cDNAs are novel and have no known homologues. Therefore, our findings argue for the sequencing of the full transcriptome of this species. This study forms the basis for functional genomic research of *L. stagnalis *not only as a model system for various areas of neuroscience research but also for general evolutionary and comparative genomics.

## Results

### Overall statistics of the *L. stagnalis *cDNA library

**Figure 2:**
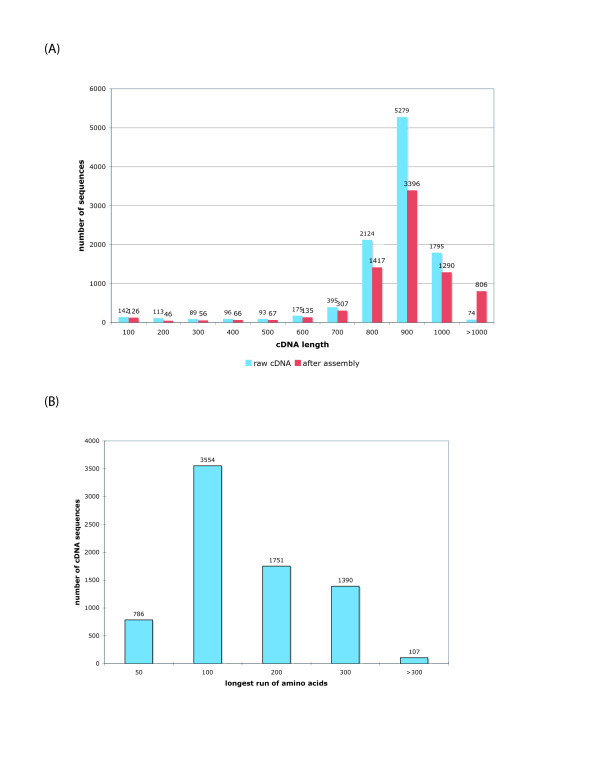
**Overall statistics of the *Lymnaea *cDNA library**.(A) Distribution of the sequence length of the cDNA sequences before and after assembly by the CAP3 program. (B) Distribution of the maximum length of translated peptide sequences.

Double-end sequencing was performed on 5376 clones that were randomly selected from the *L. stagnalis *CNS normalized library. After deleting the vector sequences, we successfully obtained a total of 10,375 EST sequences (GenBank EST Acc. number: ES291075 - ES291826; ES570937 - ES580561), which cluster to 7,712 unique sequences. The median length of the sequence reads was 838 nucleotides (mean 803 nt), the shortest being 20, and the longest cDNA being 1044 nucleotides. In fact, close to 90% of the raw sequences (9272) are longer than 700 nucleotides (Figure 2A). The mean and median G+C content of the cDNA library is 36%.

### Post-processing and sequence assembly

To estimate the level of sequence redundancy and overlap within our library, we first ran a BLAST search against the raw sequence library itself, and noticed that a large number of sequences overlap, most likely as the result of bi-directional sequencing. We then assembled the cDNA sequences by using the CAP3 software [39], which identified 6,139 singlet sequences, and 1,573 clusters (default parameters were used as suggested by the authors of CAP3), yielding 7,712 sequences with unique cDNA sequences. The median length of these assembled cDNAs is 847 nucleotides (with mean of 862 nt), the shortest being 20 nucleotides, and longest being 3,227 nucleotides. Figure 2A compares the distribution of the sequence length before and after the sequence assembly. We next translated the 7,712 assembled cDNA sequences into six possible open reading frames (ORFs), and selected the largest ORF for each sequence. Figure 2B shows that 3,248 cDNA sequences contain ORFs that are longer than 100 amino acids, which is a strong indication that these cDNAs cover protein coding regions.

### Comparison with previously published L. stagnalis EST sequences

Previously, Davison and Blaxter [33] sequenced a small set of ESTs from unnormalized libraries of *L. stagnalis *CNS and the EST set contained 1,320 sequences with an average length of 665 nt. Using these data as query we searched for homology hits in our cDNA library. Using a stringent cutoff requiring sequence identity of at least 90% and a minimum alignment length of 30 nucleotides, 509 (38.6%) sequences in our library have a hit in the previous library, and 711 (54%) of the sequences in the earlier library have a hit in ours. These differences may be accounted for 1) by the sizes of the libraries used, and 2) the previous ESTs may include redundant sequences.

### Comparison with NCBI non-redundant protein and nucleotide sequence libraries

We next ran BLASTx searches (using translated nucleotides as a query to search against protein sequence library) and mapped the *L. stagnalis *cDNA sequences to the NCBI (; up to May, 2009) non-redundant protein library (nr), which is commonly used as the principle target database to search for protein homologies. This database was constructed by pooling the sequences from the following databases, followed by clustering and removing redundant entries: translated protein coding region of all the sequence entries in the GenBank, RefSeq database (high quality proteins from human and mouse), PDB (protein structure database), SwissProt, PIR (Protein Information Source), PRF (Protein Research Foundation).

In addition, we carried out BLAST searches against the NCBI non-redundant nucleotide sequence data base (nt), which stores all the nucleotide sequences submitted to GenBank to account for the possibility that some of the shorter cDNA sequences were derived from 3' or 5' untranslated regions (UTR) of mRNA transcripts instead of mapping to the protein coding region. This also covers cDNAs that are derived from non-coding RNA transcripts such as rRNA.

**Figure 3:**
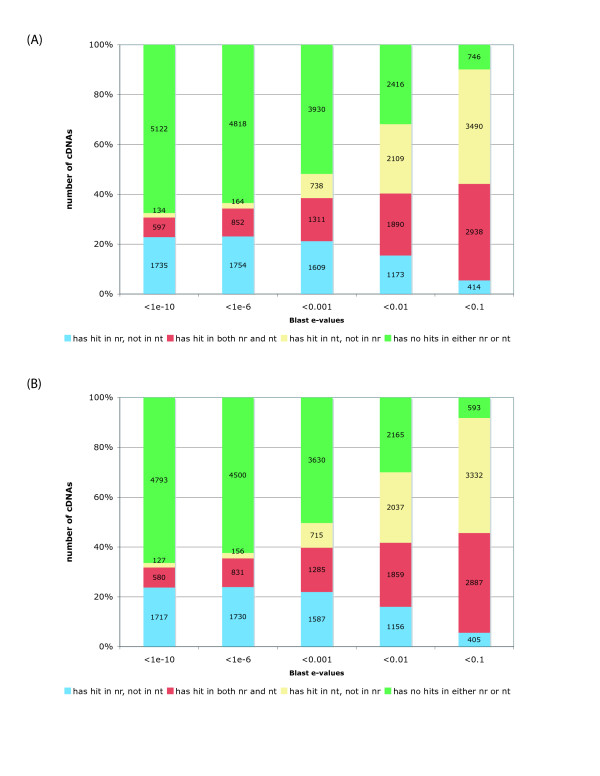
**Distribution of BLAST hits of *Lymnaea *cDNA sequences to NCBI non-redundant protein (nr) and nucleotide sequence databases (nt)**. Analyses were limited to cDNA sequences with a length of at least 100 nucleotides (A) and 500 nucleotides (B).

The results of these two BLAST searches are plotted in Figure 3. For each e-value cutoff, we divided the *L. stagnalis *sequences into four categories: (i) having a hit in "nr (protein)" but not in "nt (nucleotide)", (ii) having a hit in both "nr" and "nt", (iii) having a hit in "nt" but not in "nr", and (iv) having a hit in neither "nr" nor "nt". Our prediction was that the third category likely represents 3' or 5' UTRs or noncoding RNA transcripts. A closer inspection of the reads in category (iii) revealed that most of these reads that actually map to EST or cDNA sequences in "nt" do not have a corresponding entry in "nr", mostly because that these nt sequences are either too short or do not contain an open reading frame. The fourth category, on the other hand, likely represents novel sequences that only exist in *L. stagnalis*. However, it is also possible that some of the sequences in this category are reads from the 5' or 3' UTRs or from non-coding transcripts. The analysis was restricted to those cDNA sequences that are longer than 100 or 500 bases and the results are shown in Figure 3A and 3B.

Using an e-value cutoff of 1e-6, only 34% (2,606) of the *L. stagnalis *cDNAs had a statistically significant hit to a protein in the non-redundant database (Figure 3A). Even at an e-value cutoff of 0.001, 52% (3,930) of *L. stagnalis *cDNA sequences longer than 100 bases (Figure 3A) and 50% (3,630) of cDNA sequences longer than 500 bases (Figure 3B) have no significant BLAST matches in the non-redundant protein and nucleotide databases. This is likely caused by the relatively low coverage of DNA or genomic sequences from the Mollusca phylum in the current sequence databases, including the *A. californica *and *B. glabrata *EST databases.

We next investigated the phylogenetic distribution of the top BLAST hits. For each *L. stagnalis *cDNA, we found the phylum of its best BLAST match, and then catalogued the best matches according to their phylogenetic origin and counted the matches for each phylum. Table 1 shows the frequency of the matches with an e-value cutoff 1e-10 as used by Moroz et al (2006) [38]. The hits to chordate sequences are significantly higher than the other phyla, including Lophotrochozoan. However, this raises a question about the usefulness of such an analysis for sequence mapping as the frequency of hits is obviously associated with the number of the sequences available in the database. The number of sequences from each of the metazoan phyla in the database is largely variable as the different phyla are not all equally covered. Thus, such a presentation has the potential to introduce a serious bias towards phyla that are better presented in the databases.

**Table 1:** Phylogenetic distribution of the top hits in the NCBI nr library

**Phylogenetic group**	**Number of cDNAs** **(e-value < 1e-10)**
Chordata	944

Arthropoda (e.g. insects and Crustaceans)	562

Lophotrochozoan, including Mollusca, Annelida (segmented worms)	302

Echinodermata	273

Cnidaria (e.g. Hydra and jelly fish)	212

Nematoda (including C. elegans)	57

Platyzoa	47

Bacteria	46

Plants	41

Fungi	33

	

Total	2583

### Comparison with *A. californica *sequences

*A. californica *is currently the only other mollusk species for which an effort has been made to obtain the complete CNS transcriptome sequences [38]. To better understand the sequence diversity between these mollusk species, we compared the *L. stagnalis *cDNA sequences (7712 sequences with an average length of 862 nucleotides) with the EST sequences from *A. californica *reported by Moroz et al [38]. The published *A. californica *EST dataset includes 199,689 sequences surveyed from a wide range of tissues, with an average length of 563 nucleotides. Table 2 provides data on the cumulative populations of hits between the *L. stagnalis *and *A. californica *ESTs. We first conducted the Blastn analysis between the two datasets. At e-value cutoff of 1E-6, approximately 13% of the *L. stagnalis *cDNAs has a BLASTn hit in the *A. californica *EST library, and approximately 6% of the *A. californica *library has a match in this *L. stagnalis *cDNA library (Table 2). At a less stringent e-value cutoff of 0.01, hits for an additional ~7% of the *L. stagnalis *cDNAs were found in the *A. californica *EST library, and an additional ~7% of the *A. californica *library produced hits in this *L. stagnalis *cDNA library (Table 2). Because these two species are distantly related organisms, the low hits resulted from our Blastn analysis may be simply due to the difference in their genetic codes. We thus carried out a tblastx analysis to search a translated nucleotide database using a translated nucleotide query. As presented in Table 2, at e-value cutoff of 1e-6, the tblastx hits of *L. stagnalis *ESTs in *A. californica *database reached 28.9%, whilst the percentage of tblastx hits of *A. californica *sequences in the *L. stagnalis *EST database was 11.9%. Taking together, these data indicate a relative low percentage of matches between *A. californica *and *L. stagnalis *EST sequences.

**Table 2:** Blast hits of *L. stagnalis *cDNA sequences in the published *A. californica *EST library

**Blast** **E-value**	**Number of *L. stagnalis *ESTs having a blast hit in *A. californica***		**Number of *A. californica *ESTs having a blast hit in *L. stagnalis***	
	
	**Blastn**	**Tblastx**	**Blastn**	**Tblastx**
1e-20	523 (6.8%)	1629 (21.1%)	7,200 (3.6%)	15,801 (7.9%)

1e-10	822 (10.7%)	1987 (25.8%)	10,247 (5.1%)	20,081 (10.1%)

1e-6	981 (12.7%)	2225 (28.9%)	12,083 (6.1%)	23,704 (11.9%)

1e-3	1297 (16.8%)	2661 (34.5%)	16,823 (8.4%)	29,955 (15.0%)

0.01	1555 (20.2%)	2924 (37.9%)	26,121 (13.1%)	37,721 (18.9%)

0.1	3311 (42.9%)	3766 (48.8%)	80,725 (40.4%)	76,575 (38.3%)

				

Total	7712		199,689	

Comparison of blastn (search a nucleotide database using a nucleotide query) and tblastx searches (search translated nucleotide database using a translated nucleotide query).

It is possible that some of these *L. stagnalis *cDNAs are not expressed at high levels in *A. californica *and are not detectable in the transcriptome studies; therefore we compared the *L. stagnalis *cDNA sequences against the *A. californica *genomic trace files, downloaded from NCBI GenBank. At the e-value cutoff of 1e-6, about 13% of the *L. stagnalis *cDNAs have a hit in the *A. californica *trace library (Table 3). Another possible explanation for the relatively low match between *L. stagnalis *and *A. californica *could be that a large proportion of corresponding ESTs match to different parts of the same (orthologous) mRNAs and are therefore not identified as a match. To test this we took the 1,074 *L. stagnalis *ESTs with a best scoring match to NCBI nr and ran the respective NCBI nr match sequences against the *A. californica *EST database using a tBLASTx search. We expected that orthologous non-overlapping ESTs from both species would thus be bridged by their corresponding longer NCBI nr sequence match. Surprisingly only ~0.2% (2 out of 1074) of the *L. stagnalis *ESTs could be matched with an *A. californica *EST with an e-value ≤ 0.01. Although there are several limitations with respect to comparing EST datasets, especially when they are relatively small in size, these findings together seem to suggest that there is high sequence divergence between *A. californica *and *L. stagnalis*.

**Table 3:** Blast hits in the *A. californica *trace library using the *L. stagnalis*cDNA as query

**Blast E-value**	**Number of hits**	**%**	**cumulative**	**%**
1.E-20	264	3.42%	264	3.42%

1.E-10	405	5.25%	669	8.67%

1.E-06	327	4.24%	996	12.91%

1.E-03	455	5.90%	1,451	18.81%

0.01	450	5.84%	1,901	24.65%

0.1	1,622	21.03%	3,523	45.68%

1	3,387	43.92%	6,910	89.60%

>1	802	10.40%	7,712	100.00%

Sum	7,712			

### Comparison with *Biomphalaria glabrata *sequences

The freshwater snail *Biomphalaria glabrata (B. glabrata) *belongs to the family Planorbidae of the pulmonate gastropod mollusk. Recently the whole genome sequencing of B. glabrata has been completed , however, the trace sequences have not yet been assembled. Currently, there are 19,523 EST sequences of various B. glabrata tissues, not limited to the CNS, deposited into the GenBank. We thus compared the *B. glabrata *EST sequences with our *L. stagnalis *EST sequences. Our blastn and tblastx analyses are shown in Table 4. At e-value cutoff of 1e-6, the rate of Blastn hits of *L. stagnalis *ESTs in *B. glabrata *database was ~5% and the rate of tblastx hits was ~11%. In contrast, the Blastn hits of *B. glabrata *ESTs in our *L. stagnalis *database was ~12% and the tblastx hits were ~17%. These results indicate 1) that there is a significant level of redundancy in the *B. glabrata *EST library because many *B. glabrata *ESTs match to the same *L. stagnalis *sequences; 2) there is a relative low match between *B. glabrata *and *L. stagnalis *EST sequences, suggesting a substantial difference between the transcriptomes of the two animals.

**Table 4:** Blast hits in the B. glabrata EST library using the *L. stagnalis*cDNA as query

**Blast E-value**	**Number of *L. stagnalis *ESTs having a blast hit in *B. glabrata *library**		**Number of *B. glabrata *ESTs having a blast hit in *L. stagnalis***	
	
	**Blastn**	**Tblastx**	**Blastn**	**Tblastx**
1e-10	307 (4%)	682 (8.8%)	2098 (10.7%)	2689 (13.8%)

1e-6	375 (4.9%)	868 (11.3%)	2372 (12.1%)	3378 (17.3%)

1e-03	594 (7.7%)	1288 (16.7%)	3244 (16.6%)	4891 (25.1%)

### Comparison with other model organisms

**Figure 4:**
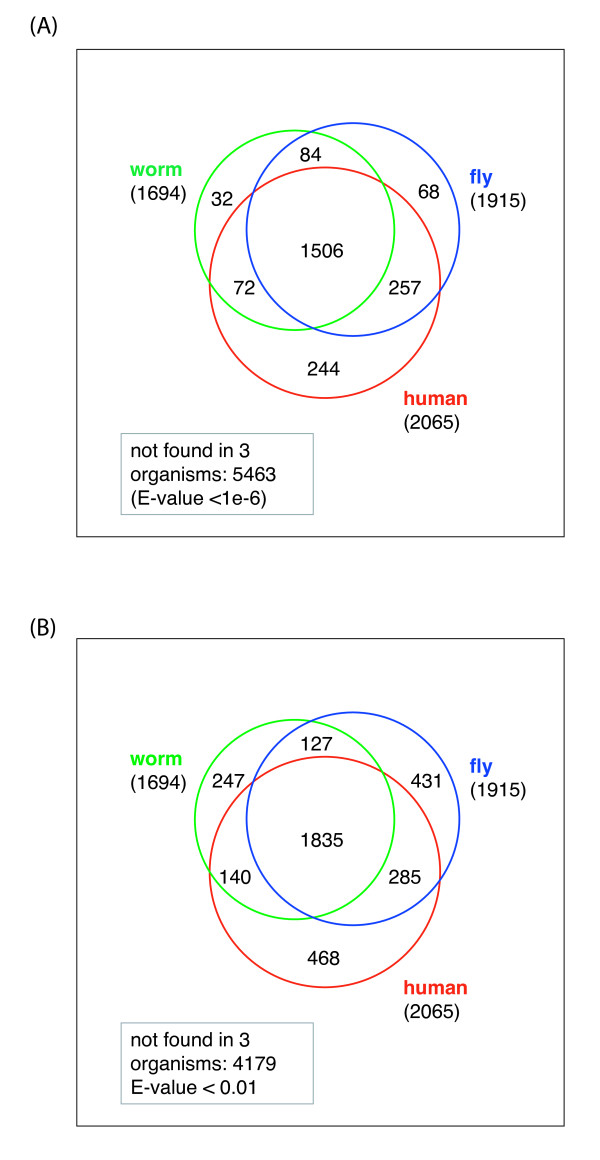
**Distribution of BLAST hits of *Lymnaea *cDNA sequences in other model organisms**. The Venn diagram shows the occurrence of BLAST hits in the protein, mRNA and noncoding RNA sequences in worm (C. elegans), fruit fly (D. melanogaster) and human, at two different BLAST e-value cutoffs. (A) e-value < 1e-6, (B) e-value < 0.01.

We investigated the occurrence of the *L. stagnalis *cDNAs in three different organisms: the model organisms nematode worm (*C. elegans*) and fruitfly (*D. melanogaster*), and human. We ran BLAST searches against the combined protein, mRNA, and noncoding RNA libraries in worm, fruitfly and human respectively; the results are shown in Figure 4. As seen in Figure 4A, with an e-value cutoff at 1e-6, 1,694 *L. stagnalis *cDNA sequences have a BLAST hit to a protein, an mRNA, or a RNA sequence in worm; 1,915 cDNAs have a hit in fly, and 2,065 cDNAs have a hit in human. Remarkably, 70% (5463) of the cDNAs do not have any BLAST hit in any of these three organisms. Similar results are shown in Figure 4B, with a BLAST cutoff at 0.01. In this case, 54% (4,179) of the cDNA sequences do not have any BLAST hit. This relatively low concordance reflects the substantial evolutionary distance between *L. stagnalis *and the three species from different animal phyla.

### Gene Ontology Mapping

We mapped the cDNA sequences to the UniProt protein database, since this is the only comprehensive database for which GO annotation data is available. Using the e-value cutoff of 1e-6, 2,242 (29%) sequences can be mapped to a UniProt entry; using an e-value of 1e-10, 2,038 (26%) can be mapped to a UniProt entry. We identified the corresponding Gene Ontology entries mapped to GOslim terms by using the tools provided by the GOA website . Table 5 shows the most frequently occurring GO functional categories.

**Table 5:** GO category distribution of the *Lymnaea *cDNAs

**GO terms**	**GO terms**	**cDNA frequency**
GO:0005515	protein binding	723
GO:0016787	hydrolase activity	350
GO:0016740	transferase activity	237
GO:0003824	catalytic activity	221
GO:0016491	oxidoreductase activity	187
GO:0005198	structural molecule activity	138
GO:0016874	ligase activity	90
GO:0030234	enzyme regulator activity	78
GO:0030528	transcription regulator activity	64
GO:0005215	transporter activity	51
GO:0015075	ion transmembrane transporter activity	45
GO:0004871	signal transducer activity	45
GO:0016829	lyase activity	44
GO:0004872	receptor activity	44
GO:0045182	translation regulator activity	42
GO:0016853	isomerase activity	38

Total		2406

### Alignment of known presynaptic genes

**Figure 5:**
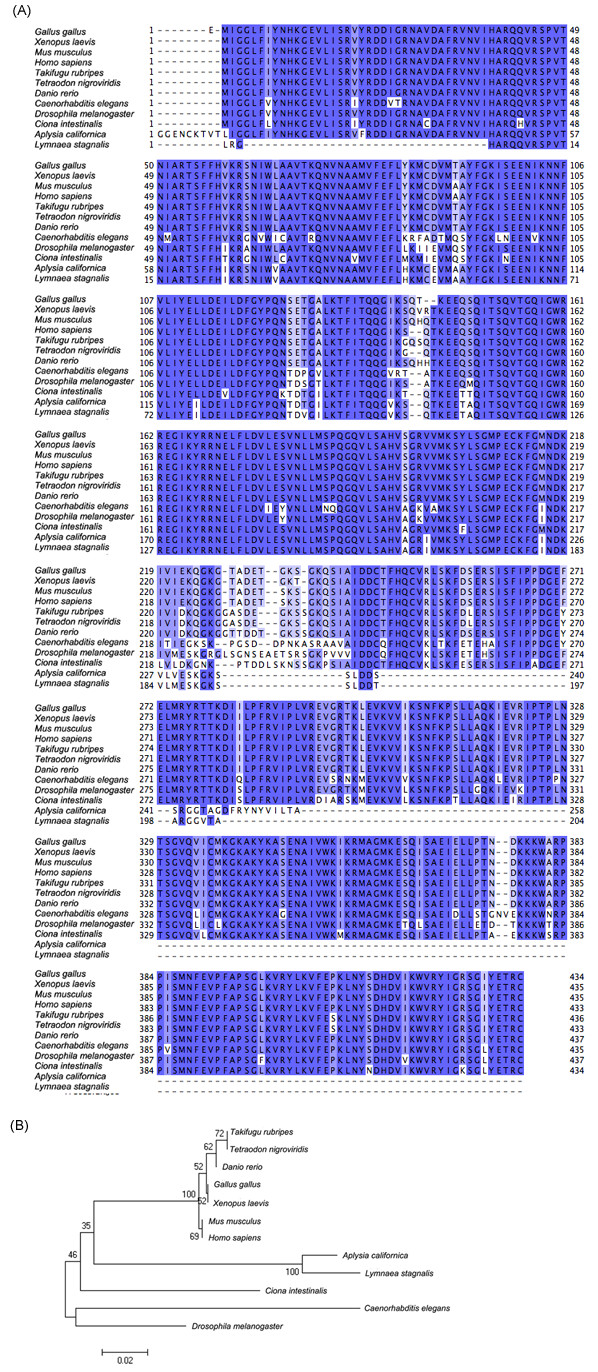
**Protein sequence alignment and phylogenetic tree of clathrin adapter-protein-2 (AP-2) mu-1 subunit**. The alignment (A) and the phylogenetic tree (B) were generated by Clustalw. FPS013.CR.J08 is the *Lymnaea *orthologue identified in this study.

**Figure 6:**
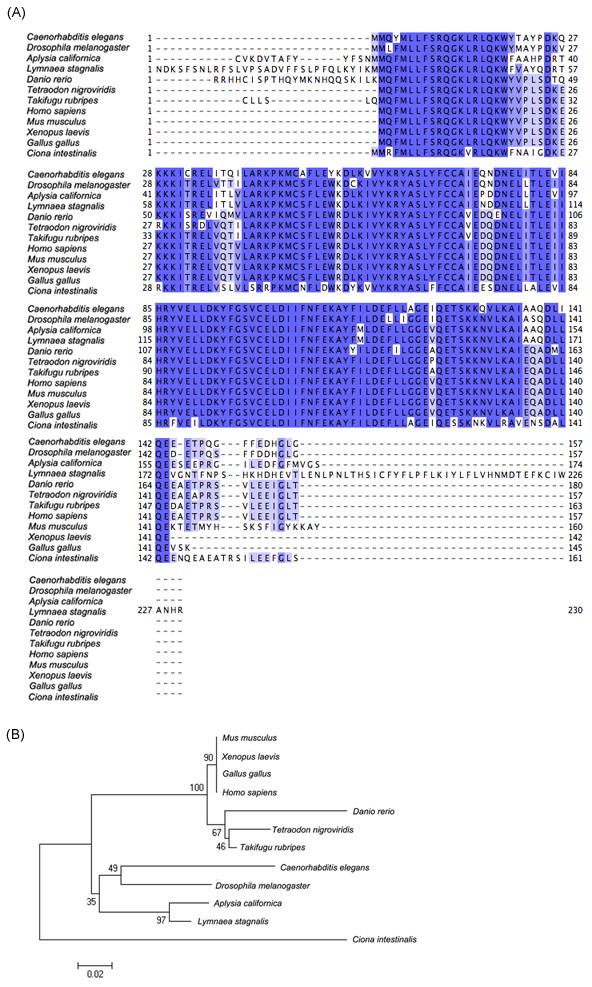
**Protein sequence alignment and phylogenetic tree of clathrin adapter-protein-1 (AP-1) theta-1 subunit**. The alignment (A) and the phylogenetic tree (B) were generated by Clustalw. FPS0112.CR.F02 is the *Lymnaea *orthologue identified in this study.

**Figure 7:**
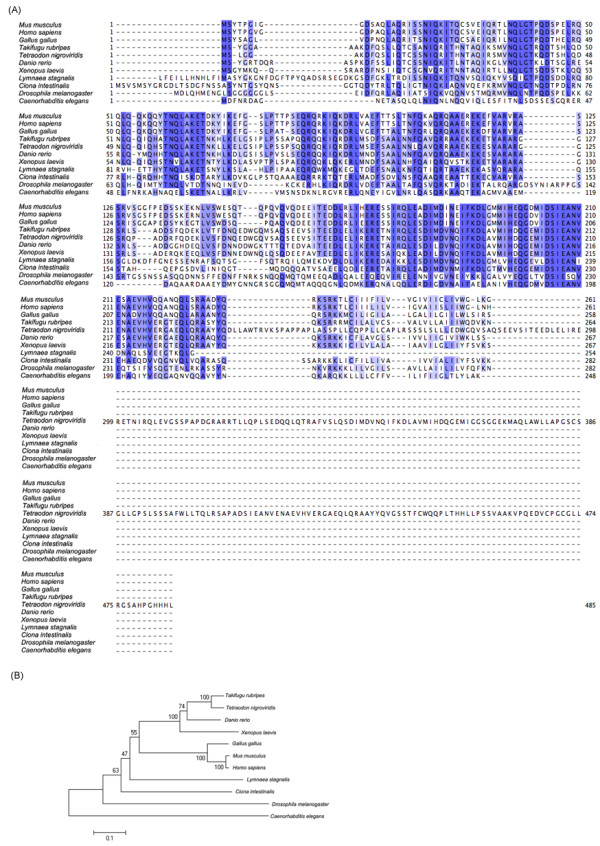
**Protein sequence alignment and phylogenetic tree of syntaxin 7**. The alignment (A) and the phylogenetic tree (B) were generated by Clustalw. FPS0110.CR.A09 is the *Lymnaea *orthologue identified in this study.

We identified a number of genes from the *L. stagnalis *library that are functionally related to presynaptic activity and other neuronal functions. We then generated multiple sequence alignments of these *L. stagnalis *proteins with their orthologous genes from other species and generated the corresponding phylogenetic trees (Figure 5, 6, and 7). We found that, as expected, the *L. stagnalis *sequences are most closely related to their orthologs in *A. californica*. Typical examples are the clathrin adaptor-protein complex 2 (AP-2) mu-1 subunit and the clathrin AP-1 theta-1 subunit shown in Figure 5 and Figure 6, respectively. The phylogenetic trees clearly show the conservation of these proteins across species. The *L. stagnalis *orthologue is more closely related to *A. californica *than other invertebrates and is distant from vertebrates. Intriguingly, *L. stagnalis *has an ortholog of the vertebrate syntaxin 7 gene, but its equivalent has so far not been found in the *A. californica *library (Figure 7).

## Discussion

In summary, we reported in this study the CNS expressed cDNA sequences of 5376 randomly selected clones from a normalized CNS cDNA library, thus providing what is currently the largest database of *L. stagnalis *cDNA sequences. Because molluscan species have not been frequently used in the sequencing projects, this is currently the second largest CNS transcriptome study in the entire Mollusca phylum. Although the transcriptome information is far from complete, we found only ~25% BLAST hits of *L. stagnalis *cDNA sequences in *A. californica *sequences, which suggests substantial genetic differences between these two gastropod mollusks. This argues for further large scale sequencing of *L. stagnalis *that will shed light on the extent of diversification of the genome in both molluscan species.

### Normalized cDNA library

In this study we used a normalized cDNA library to reduce the high variation among the abundant clones, thus increasing the probability of sequencing rare transcripts [40]. We found that over 90% of the sequences are between 700 and 1000 bp long, indicating the overall good quality of the sequencing processes. We have sequenced 5376 clones and obtained 10,375 EST sequences which cluster to 7,712 unique sequences. The comparison between our library and the previous published *Lymnaea *EST sequences generated from a non-normalized library [33] showed that 509 (38.6%) sequences in our library have a hit in the previous library, and 711 (54%) of the sequences in the earlier library have a hit in ours. The higher percentage of hits using the previous library to search the present ESTs is likely due to that 1) the previous sequencing of an unnormalized EST library includes a large proportion of redundant sequences; and 2) this study sequenced ESTs from normalized libraries which reduces the probability of redundant sequences, and thus yielded a higher coverage. In addition, the previous library comprised sequences from the 5' end of transcripts, whereas the present EST database includes a mix of 5' and 3' sequences. It thus is also possible that a proportion of sequences of the previous ESTs generate more than one hit in our database.

### Novel transcripts in *L. stagnalis *

It is intriguing that a large proportion of the *L. stagnalis *cDNAs contain novel sequences that apparently have no significant match in any of the existing databases. In fact, as shown in Figure 3B, even after restricting analyses to sequences that are longer than 500 nucleotides and a less restrictive BLAST e-value cutoff of 1e-6, approximately half of the cDNAs (3,630) have no homologous sequences in the existing databases. Many of these novel transcripts can also be translated into long sequences of amino acids. For example one cDNA (Contig 596; Supplementary Material, ) has 3216 nucleotides containing 505 consecutively translated amino acids. Interestingly, the closest sequence homolog for this sequence is a RNA polymerase in potato (gi|68124015|emb|CAJ01915.1| DNA-directed RNA polymerase [*Solanum tuberosum*]). Nevertheless, the large collection of gene sequences identified in this study include structural proteins, heat shock proteins, transcription factors, ion channels, receptors, and protein kinases.

### Alignment and phylogenetic trees

We have aligned a number of presynaptic protein sequences from *L. stagnalis *to those of other model organisms. The *L. stagnalis *presynaptic protein sequences tested here are highly homologous to other species, consistent with the notion that both invertebrates and vertebrates share similar molecular mechanisms of synaptic transmission (see [41]). Interestingly, we identified a syntaxin 7 sequence in the *L. stagnalis *CNS transcriptome. Syntaxin 7 is one of the endosomal SNARE (soluble N-ethylmaleimide-sensitive factor attachment protein receptors) proteins that may be involved in a conserved late endosomal fusion pathway [42,43]. Notably, this protein has so far not been reported in *A. californica*.

Our results demonstrate that the *L. stagnalis *CNS transcriptome data complement the transcriptome sequencing project of *A. californica *[38]. Based on the *A. californica *CNS sequence data, we expect a total of ~20,000 neuronal gene products in the *L. stagnalis *CNS. We currently obtained 2,406 transcripts corresponding to known biological processes. These annotated transcripts contribute 12% of the predicted transcriptome size. The hits of *L. stagnalis *sequences to the *A. californica *transcriptome are ~29% (e-value: 1E-6; tblastx), and an additional 9% were found to match *A. californica *sequences with an e-value cutoff at 0.01. Amongst 7712 *L. stagnalis *sequences tested, over 97% (7484 out of 7712 sequences) of them have lengths greater than 300 nucleotides, and over 42% (3248 out of 7712) sequences contain ORFs that are longer than 100 amino acids. Assuming that the *A. californica *EST set has a good coverage [38], it is a somewhat low match which indicates the possibility that the CNS transcriptome sequences of the two species are considerably different. Although both species are classified as heterobranches [44] within the gastropods, the two branches diverged over 600 million years ago. Thus it is not surprising to see these apparently large transcriptome sequence variations between these two species. However, our sequencing effort comprises only a fraction of the neuronal transcriptome and a larger scale sequencing project is required in order to make a full comparison between these two species.

## Conclusion

This study currently provides the largest database of *L. stagnalis CNS EST sequences, and demonstrates the *substantial genetic differences between two gastropod mollusks, *L. stagnalis *and *A. californica*. This study establishes a firm basis for functional genomic research in this species, for comparative and environmental genomics, and for identification of novel proteins important for neuronal functions, thus directly supporting and advancing ongoing functional work in this and related model systems.

## Methods

### Animals

*L. stagnalis *were obtained from a culture at the VU University, Amsterdam, and were raised and maintained in aquaria at the University of Calgary, as previously described [13,45]. Animals were kept in water at 20°C under a 12 hr light/dark cycle, and fed green leaf lettuce twice a week. Two-month old snails having shell lengths of 15 to 20 mm were used in this study. Specifically, the snails were anesthetized with 10% (v/v) Listerine for 10 min. The central ring ganglia and attached buccal ganglia were dissected out and prepared for total RNA extraction. Three central ring ganglia were used for total RNA extraction.

### cDNA synthesis and cDNA library normalization

cDNA synthesis and normalization were carried out by Bio S&T Inc. (Montreal, QC, Canada). Specifically, total RNA was extracted from snail ganglia using the modified Trizol method (Invitrogen, USA). Purification of mRNA was carried out using Oligotex mRNA kit (Qiagen, USA), and cDNA synthesis using the SMART™ cDNA library construction kit (Clontech, USA). The clones contain SfiI-A and SfiI-B restriction sites, which allowed directional cloning. Full-length cDNA was synthesized with two set of primers for driver and tester cDNA. Single-stranded cDNA was used for hybridization instead of double-stranded cDNA [40]. Excess amount of sense-stranded cDNA was hybridized with antisense-stranded cDNA. After hybridization, duplex structures were removed by hydroxyapatite chromatography. Normalized tester cDNA was re-amplified with the tester specific primer L4N without amplification of the driver cDNA that cannot be amplified using the L4N primer.

cDNA normalization efficiency was strictly monitored by Bio S & T Inc. (Montreal, QC, Canada). Specifically a parallel normalization of an internal control was performed in addition to the normalization of the cDNA population. In the parallel normalization, a modified reporter gene (chloramphenicol resistance gene with same adaptor-primer sequences) was added to the cDNA population before normalization at a desired redundancy rate (e.g. 1.0%) as a control. cDNA was cloned using a modified pBluescript SK-vector (MBI-Fermentas, Canada). Because pBluescript is ampicillin resistant, the percentage of this control gene is determined before and after normalization. The frequency of chloramphenicol resistant clones was determined and used to estimate the reduction during normalization. In this study, reduction was 40×.

Following normalization, ss-cDNA were amplified by PCR and purified. Re-amplified cDNAs were then subjected to SfiI digestion and size fractionation in a 1% agarose gel. cDNA fragments larger than 0.5 Kb were purified for cloning. The cloning vector was a modified pBluescriptII SK-with SfiI A&B inserts between EcoRI and XhoI. The ligated cDNAs were then transformed into DH10B (Invitrogen, USA). The clones were arrayed in a one clone/well format and amplified by overnight culture. The average insert size was 1.5 kb based on a test sample of 20 random clones. cDNA clones were prepared as glycerol stock in 384-well plates for sequencing.

### DNA Sequencing

Double-end cDNA sequencing was carried out by the Genome Sciences Centre at the British Columbia Cancer Agency (Vancouver, BC, Canada) using the Sanger sequencing method. For sequencing of 5'-ends, T3 (or M13F) primer was used; for sequencing of 3'-ends, T7 (or M13R) primer was used. The sequences listed are all in the forward strand, 5' to 3'.

### Sequence analysis and bioinformatics

The raw cDNA sequences were subjected to 5'-trimming to eliminate the vector and 3'-trimming to eliminate low quality portions. The sequences were then clustered and assembled into 7712 sequences using the program CAP3 [39]. We used the BLAST program [46] to search for homology sequences in the following sequence databases: NCBI non-redundant protein (nr) and nucleotide (nt) databases, Swissprot protein database, and EST databases including the previously published EST library of *A. californica *[38]. We wrote PERL scripts to conduct the subsequent bioinformatic analyses (available upon request).

## Authors' contributions

ZPF and NN carried out the molecular genetic studies; ZZ conducted the bioinformatics analysis; ZPF, ZZ, and NN participated in the sequence alignment; ZPF and ZZ drafted the manuscript; ZPF, ZZ, REVK, VAS, RPC, and ABS participated in producing the final version of the manuscript. KJ developed the sequence database. ZPF, REVK, NIS, and ABS participated in the design of the study. ZPF, NIS and ABS conceived of the study, and ZPF coordinated the study. All authors read, edited and approved the final manuscript.
